# Determinants of health-related quality of life in adults with cystic fibrosis: the role of selected sociodemographic and treatment-related factors

**DOI:** 10.1186/s13023-026-04362-x

**Published:** 2026-04-24

**Authors:** Dorota Snop-Perkowska, Jakub Świtalski, Edyta Krzych-Fałta, Anna Augustynowicz

**Affiliations:** 1 School of Public Health, Centre of Postgraduate Medical Education of Warsaw, Warsaw, 01-826 Poland; 2 National Tuberculosis and Lung Diseases Research Institute, Warsaw, 01-138 Poland; 3 Department of Ethics and Medical Law, Center for the Humanities and Social Sciences in Medicine, Medical University of Warsaw, Litewska 14a St, Warsaw, 00-581 Poland; 4 Department of Nursing Propedeutics, Faculty of Health Sciences, Medical University of Warsaw, Warsaw, 01-445 Poland; 5 VIZJA University, Warsaw, 01-043 Poland

**Keywords:** Cystic fibrosis, Quality of life, Health status, Adult, Poland

## Abstract

**Background:**

In recent years, advances in modern, effective therapies have significantly increased the life expectancy of patients with cystic fibrosis (CF). However, this increase presents new challenges. It is becoming increasingly important to measure the health-related quality of life (HRQoL) and the factors that influence it in this patient population. This study aims to assess the HRQoL of adult patients with CF in Poland and to analyse the sociodemographic and health factors that affect it.

**Methods:**

A cross-sectional study was conducted online using the Polish version of Cystic Fibrosis Questionnaire-Revised 14+ (CFQ-R 14+) between January and May 2024. The questionnaire included also sociodemographic questions such as gender, age, marital status, education level, place of residence, and educational or professional activity. Additionally, it included questions on health and treatment, such as pain level, comorbidities, and the use of cystic fibrosis transmembrane conductance regulator (CFTR) modulators. Various statistical methods were employed, including Student’s t-test for independent samples, the Kruskal-Wallis test, and correlation analyses, to assess the impact of individual factors on HRQoL.

**Results:**

The analysis included responses from 220 individuals across several centers in Poland. The average age of the respondents was 30.56 years (min = 18, max = 61). The highest-rated aspects of HRQoL were related to food-related (eating, digestive symptoms), while the lowest ratings were given for vitality, emotional functioning, and health perceptions. Generally, individuals who were educationally or professionally active, those taking CFTR modulators, and those experiencing less pain reported a better HRQoL across almost all domains. Factors such as gender, marital status, education, and comorbidities influenced some aspects of HRQoL. In contrast, other factors, such as age and place of residence, did not affect patients’ HRQoL.

**Conclusions:**

The HRQoL of adult patients with CF in Poland varies across assessed domains and is significantly influenced by both health and social factors. Integrating psychological and social support into treatment may help further improve patients’ HRQoL.

**Supplementary Information:**

The online version contains supplementary material available at 10.1186/s13023-026-04362-x.

## Background

Quality of life (QoL) is a multidimensional concept referring to an individual’s overall perception of their position in life within their cultural and value context [1]. Health-related quality of life (HRQoL) represents a narrower construct, focusing specifically on the impact of health status, disease, and treatment on physical, psychological, and social functioning [2]. Ensuring a good HRQoL for patients dealing with chronic diseases is a primary concern for medical staff and researchers alike [3]. In some cases, the impact on HRQoL is so profound that enhancing this aspect becomes a central goal of care, alongside improving clinical outcomes [4, 5].

Cystic fibrosis (CF), a rare disease, can significantly deteriorate HRQoL due to its various symptoms [6]. Even with substantial advances in treatment – particularly the introduction of modern therapies like CFTR (cystic fibrosis transmembrane conductance regulator) modulators – the disease continues to pose challenges for patients and demands ongoing medical oversight [7]. While it’s undeniable that CFTR modulators have led to significant increases in life expectancy for those with CF, simply prolonging life without enhancing its quality cannot be deemed a true success [8]. Therefore, it is vital to consider the daily functioning and challenges faced by patients who are living longer and navigating new challenges in their lives [9].

It is estimated that up to 190,000 people worldwide may have CF (of whom about 50,000 are in Europe) [10]. There is a lack of precise information regarding the number of people living with CF in Poland. According to data from the European Cystic Fibrosis Society Patient Registry (ECFSPR), the number of CF patients in Poland is estimated at approximately 1,700 (of whom about 40% are adults) [11]. Meanwhile, the National Health Fund (Poland’s public healthcare payer) estimates the number of adult patients at around 1,000 (based on the number of healthcare services provided) [12].

The HRQoL for patients with chronic conditions like CF is a pressing issue that demands attention. Due to its ongoing and progressive nature, CF impacts various aspects of patients’ daily lives. Prolonged treatment, frequent hospitalisations, physical limitations, perceived pain and the necessity of following complicated, time‑consuming therapy regimens (including daily physiotherapy, inhaled and oral medications, supplementation, dietary management, and regular clinical monitoring) can contribute to diminished mental well-being, social isolation, and a decrease in professional activity [13–16]. Several factors can influence how individuals perceive their QoL, including health-related factors (such as pain levels and comorbidities) and sociodemographic characteristics (such as gender, age, and marital status) [17, 18]. Analysing these factors is crucial for clinical practice and therapeutic support, as it helps identify groups that may be particularly at risk of decreased well-being and highlight areas in need of intervention. This understanding can also serve as a foundation for planning educational initiatives and interventions to enhance the HRQoL and optimize care for patients with CF [19, 20].

Studies on the HRQoL for those with CF have been conducted in Poland, but typically within small patient populations, fewer than 100 individuals [21–26]. Additionally, these studies usually recruited patients from a single centre, limiting the ability to draw broader conclusions. Furthermore, the results have often combined data from children, adolescents, and adults, complicating the examination of the distinct needs of these groups. It’s important to note that children and adolescents have different needs and perceptions of their HRQoL than adults do. Some of these studies were also conducted roughly a decade ago, before the widespread adoption of modern treatments like CFTR modulators [21–23].

This publication is part of a research project that conducts a detailed analysis of the HRQoL of adult patients with CF in Poland. Our previously published articles have also examined this patient population, specifically regarding the psychometric properties of the Polish version of the Cystic Fibrosis Questionnaire-Revised 14+ (CFQ-R 14+), as well as the impact of certain factors on the severity of depression and generalised anxiety among these patients [27, 28]. Additionally, we analysed the psychometric properties of the Patient Health Questionnaire-9 (PHQ-9) and the General Anxiety Disorder-7 (GAD-7) within this population [28]. This publication is the final entry in our series, aiming to assess the HRQoL of adult patients with CF in Poland and to analyze specific sociodemographic and health factors that may influence it.

## Methods

### Study design

This cross-sectional study was conducted between January and May 2024, using an online format across various centres in Poland, with support from organizations representing Polish patients with CF. It exclusively included responses from patients aged 18 and older who were diagnosed with CF, identified as Polish citizens, and fluent in Polish. To recruit participants, we employed a two-pronged strategy designed to maximize coverage. First, we approached major cystic fibrosis treatment centers across the country, requesting their assistance by forwarding the survey link directly to eligible patients. Also, we reached out to patient advocacy organizations representing adults with cystic fibrosis. These organizations disseminated the survey link to their networks of adult patients. This recruitment approach, combining clinical centers and patient organizations, was intended to maximize sample size. Verification of the study’s inclusion criteria was conducted by the treatment centers and patient organizations, which recruited participants by sending them email invitations encouraging them to take part in the study. This was not the only stage of verification, as we additionally checked at the data analysis level whether participants met the eligibility criteria, which were assessed through separate questions in the online questionnaire (e.g., regarding age or CF diagnosis). The detailed methodology for patient recruitment and study execution is described in a previous publication [27].

For this study, we employed the CFQ-R 14+, which we had previously analysed to evaluate its psychometric properties. We conducted a confirmatory factor analysis (CFA) followed by an exploratory factor analysis (EFA) using the parallel analysis method, principal axis factoring, with Oblimin rotation [27]. The CFA results did not confirm the initially proposed 12-factor structure within the Polish patient population (using online measures). As a result, we developed a new structure that streamlined the number of questions and domains. Instead of the original 12 domains comprising 50 questions, we created 6 domains (Physical; Vitality & Emotion; Treatment Burden & Health Perceptions; Respiratory; Eat, Weight, Body; Digestion) totaling 40 questions. The most consistent domains were “Respiratory” and “Digestion”, which retained the same questions as those in the original version of the tool. Details regarding the new factor structure can be found in a previously published article [27].

To measure pain levels, we chose the Numerical Rating Scale (NRS). Patients were asked to rate their average pain experienced over the past two weeks on a scale from 1 (the lowest possible pain level) to 10 (the highest possible pain level).

### Variables and statistical analysis

The sociodemographic and health data of the study participants were first presented, including age, gender, education, comorbidities, and pain levels. Next, we calculated the CFQ-R 14 + scores across various domains using the original scale, presenting both raw and rescaled results (supplementary material, Table S1). Although the tool’s factor structure had not been confirmed in the Polish adult population, we opted to calculate these scores, as they are widely used in studies both in Poland and globally. Subsequently, we calculated the scores taking into account the revised structure described above. We presented the results for each scale by applying the transformation formula from the original CFQ-R tool: (sum of responses - minimal possible sum) / (maximum possible sum - minimal possible sum) × 100.

Normality of the data was evaluated using the Kolmogorov-Smirnov test, ensuring that assumptions for subsequent parametric tests were met. For subsequent analyses, we opted for the new factor structure without modifying the results on a scaled basis. To address outliers, the winsorization method was employed, which replaces extreme values in the dataset with predetermined limits. In our case, we adopted the last data point preceding the extreme value. This approach allows us to retain observations with extreme values in the sample while significantly reducing their impact on descriptive statistics.

All calculations described below were based on the raw data. The Student’s t-test for independent samples was used to examine the relationship between CFQ-R 14 + results and factors such as marital status, gender, the presence of comorbidities, and the use or non-use of CFTR modulators. Where Levene’s test indicated significant variance, we reported the results with Welch’s correction.

We also conducted correlation analyses using Pearson’s r for age and perceived pain, and Spearman’s rho for education level and respondents’ place of residence. Additionally, we employed the Kruskal-Wallis test to evaluate how CFQ-R 14 + scores varied across educational or professional situation over the past two weeks. To determine specific differences between groups, we applied a post hoc test with Dunn-Bonferroni correction.

All calculations were performed using IBM SPSS Statistics (version 29). The level of statistical significance was set at α = 0.05. We adopted the following effect sizes for individual coefficients: Cohen’s d (weak < 0.5, moderate 0.5–0.7, and strong ≥ 0.8) [29]; correlation (weak ≤ 0.3, moderate 0.4–0.6, and strong ≥ 0.7) [30]; η² (weak < 0.06, moderate 0.06–0.13, and strong ≥ 0.14) [29].

## Results

### Characteristics of the study group

The study involved 220 Polish adults diagnosed with CF, with an average age of 30.56 years (min = 18, max = 61). Among the participants, women accounted for 59.1% (*n* = 130), while men accounted for 40.9% (*n* = 90). A significant portion of the respondents had either secondary education (*n* = 103; 46.8%) or higher education (*n* = 101; 45.9%). The participants’ places of residence varied widely, with many living in large cities of over 250,000 inhabitants (*n* = 69; 31.4%), as well as in rural areas in rural areas (*n* = 58; 26.4%). Notably, 64.5% (*n* = 142) of the study group reported being single or not in a relationship.

Moreover, over half of the respondents reported comorbidities (*n* = 132; 60%). Among the reported comorbid conditions, diabetes was the most prevalent (*n* = 58). Additional health problems reported by respondents included disorders of the digestive system (such as celiac disease), thyroid diseases, asthma, sinus diseases, liver cirrhosis, and depression. A considerable number of patients were receiving treatment with CFTR modulators (*n* = 158; 71.8%). When asked about pain levels over the past 2 weeks, the average NRS score was 2 (the minimum value indicated by the respondents = 1, the maximum value indicated by the respondents = 9). Detailed information regarding the sample can be found in a previously published study [27].

### Patients’ health-related quality of life based on CFQ-R 14 + scores

We derived the following results for each domain: Physical (68.44); Vitality & Emotion (58.26); Treatment Burden & Health Perceptions (62.62); Respiratory (73.23); Eat, Weight, Body (76.16); Digestion (73.28). The raw CFQ-R 14 + scores based on the new factor structure obtained in our study are presented in Table 1. The calculations presented below were based on the raw data. The Kolmogorov-Smirnov test indicated significant differences in the distributions of the studied variables compared to a normal distribution. Nevertheless, the skewness and kurtosis values after transformation ranged from − 2 to 2, suggesting that the distributions approached normality [31]. Consequently, we performed further analyses using parametric methods, provided that the other assumptions of these tests were met.

**Table 1:** Descriptive statistics and Kolmogorov-Smirnov test results for the domains of the Polish adaptation of the CFQ-R 14 + among adult patients

Domain	M	Me	SD	Sk	Kurt	Min.	Max.	D	*p*
Physical	30.53	33.00	7.49	-0.81	-0.09	10.00	40.00	0.14	< 0.001
Vitality & Emotion;	21.98	22.50	5.24	-0.40	-0.34	9.00	32.00	0.09	< 0.001
Treatment Burden & Health Perceptions	20.15	20.00	4.29	-0.34	-0.44	9.00	28.00	0.09	< 0.001
Repiratory	19.18	20.00	4.05	-0.70	-0.47	8.00	24.00	0.15	< 0.001
Eat, Weight, Body*	19.72	20.00	3.81	-0.63	-0.48	9.00	24.00	0.14	< 0.001
Digestion*	9.60	10.00	1.88	-0.74	0.33	4.00	12.00	0.14	< 0.001

D – Kolmogorov-Smirnov test result; Kurt – kurtosis; M – mean; Max – maximum value; Me – median; Min – minimum value; SD – standard deviation; Sk – skewness

*Variables after winsorized transformation. Number of outlier observations: Eat, Weight, Body (*n* = 1); Digestion (*n* = 1)

### Determinants of health-related quality of life among Polish adult patients with cystic fibrosis

The results of gender comparisons revealed significant differences in the HRQoL across the following domains: Physical (t_(218)_=-3.44; *p* < 0.001; d = 0.47), Vitality & Emotion (t_(218)_=-2.80; *p* = 0.005; d = 0.38), Eat, Weight, Body (t_(218)_ = 2.35; *p* = 0.020; d = 0.32). In the first two domains, men reported a higher HRQoL than women. However, in the domains of Eat Weight, Body, women achieved higher levels than men. According to Cohen’s d index, the effect sizes were small (Table 2).

**Table 2:** Health-related quality of life of adult Polish patients with cystic fibrosis based on gender

Domain	Women (*n* = 130)		Male (*n* = 90)		t	df	*p*	95% CI		Cohen’s d
	M	SD	M	SD				LL	UL	
Physical	29.20	7.53	32.59	6.64	-3.44	218	< 0.001	-5.33	-1.45	0.47
Vitality & Emotion;	21.17	5.15	23.16	5.18	-2.80	218	0.005	-3.38	-0.59	0.38
Treatment Burden & Health Perceptions	20.05	4.14	20.30	4.53	-0.43	218	0.667	-1.42	0.91	0.06
Repiratory	18.82	4.26	19.71	3.69	-1.62	218	0.107	-1.99	0.19	0.22
Eat, Weight, Body	20.22	3.80	19.00	3.74	2.35	218	0.020	0.20	2.24	0.32
Digestion	9.44	2.01	9.84	1.64	-1.58	218	0.115	-0.91	0.10	0.22

CI – confidence interval; df – degrees of freedom; LL – lower limit; M – mean; SD – standard deviation; t – value of the Student’s t-test statistic for independent samples; UL – upper limit

Based on respondents’ marital status, only one statistically significant correlation was found. Individuals in relationships reported a higher HRQoL than single individuals in the Vitality & Emotion domain (t_(218)_ = -2.07; *p* = 0.040; d = 0.29). However, the effect observed was weak (Table 3).

**Table 3:** Health-related quality of life of adult Polish patients with cystic fibrosis based on marital status

Domain	Single (*n* = 142)		In a relationship (*n* = 78)		t	df	*p*	95% CI		Cohen’s d
	M	SD	M	SD				LL	UL	
Physical	30.47	7.12	30.64	8.16	-0.16	218	0.873	-2.25	1.91	0.02
Vitality & Emotion	21.44	5.22	22.96	5.19	-2.07	218	0.040	-2.96	-0.07	0.29
Treatment Burden & Health Perceptions	19.99	4.26	20.44	4.37	-0.73	218	0.465	-1.64	0.75	0.10
Repiratory	19.38	3.90	18.82	4.31	0.98	218	0.328	-0.57	1.68	0.14
Eat, Weight, Body	19.35	3.78	20.38	3.81	-1.93	218	0.055	-2.09	0.02	0.27
Digestion	9.53	2.01	9.73	1.63	-0.76	218	0.447	-0.73	0.32	0.11

CI – confidence interval; df – degrees of freedom; LL – lower limit; M – mean; SD – standard deviation; t – value of the Student’s t-test statistic for independent samples; UL – upper limit

In relation to the presence of comorbidities, differences were observed in the following domains: Physical (t_(218)_ = 2.59; *p* = 0.010; d = 0.36) and Treatment Burden & Health Perceptions (t_(218)_ = 2.33; *p* = 0.021; d = 0.32). Individuals who did not have comorbidities demonstrated better results in the aforementioned domains. Once again, a weak effect was observed (Table 4).

**Table 4:** Heath-related quality of life of adult Polish patients with cystic fibrosis based on the presence of comorbidities

Domain	No comorbidities (*n* = 88)		Presence of comorbidities (*n* = 132)		t	df	*p*	95% CI		Cohen’s d
	M	SD	M	SD				LL	UL	
Physical	32.11	7.19	29.49	7.48	2.59	218	0.010	0.62	4.62	0.36
Vitality & Emotion	22.61	5.26	21.56	5.21	1.46	218	0.145	-0.37	2.47	0.20
Treatment Burden & Health Perceptions	20.97	4.45	19.61	4.11	2.33	218	0.021	0.21	2.51	0.32
Repiratory	19.52	3.98	18.95	4.10	1.02	218	0.309	-0.53	1.67	0.14
Eat, Weight, Body	20.22	3.82	19.39	3.79	1.59	218	0.114	-0.20	1.86	0.22
Digestion	9.66	1.71	9.58	1.96	0.32	218	0.746	-0.42	0.59	0.04

CI – confidence interval; df – degrees of freedom; LL – lower limit; M – mean; SD – standard deviation; t – value of the Student’s t-test statistic for independent samples; UL – upper limit

In terms of the differences observed between groups who were and weren’t taking CFTR modulators, several significant findings emerged in the following domains: Physical (t_(90.85)_=-4.01; *p* < 0.001; d = 0.67), Treatment Burden & Health Perceptions (t_(218)_=-4.81; *p* < 0.001; d = 0.72), Respiratory (t_(93.85)_=-4.49; *p* < 0.001; d = 0.74) and Eat, Weight, Body (t_(218)_=-2.89; *p* = 0.004; d = 0.43). Analysis showed that those who received CFTR modulator therapy had better HRQoL in these domains than those who did not (Table 5).

**Table 5:** Health-related quality of life of adult Polish patients with cystic fibrosis based on CFTR modulator use

Domain	No use of CFTR modulators (*n* = 62)		Use of CFTR modulators (*n* = 158)		t	df	*p*	95% CI		Cohen’s d
	M	SD	M	SD				LL	UL	
Physical	27.10	8.49	31.90	6.55	-4.01ᵃ	90.85	< 0.001	-7.18	-2.42	0.67
Vitality & Emotion	20.90	5.51	22.41	5.09	-1.92	218	0.056	-3.04	0.04	0.29
Treatment Burden & Health Perceptions	18.03	3.90	20.98	4.16	-4.81	218	< 0.001	-4.16	-1.74	0.72
Respiratory	17.16	4.42	19.99	3.57	-4.49ᵃ	93.85	< 0.001	-4.08	-1.58	0.74
Eat, Weight, Body	18.53	4.18	20.17	3.61	-2.89	218	0.004	-2.75	-0.52	0.43
Digestion	9.66	1.95	9.58	1.86	0.30	218	0.763	-0.47	0.64	0.05

CFTR – cystic fibrosis transmembrane conductance regulator; CI – confidence interval; df – degrees of freedom; LL - lower limit; M – mean; SD – standard deviation; t – value of the Student’s t-test statistic for independent samples; UL – upper limit

* Levene’s test was statistically significant – the result was reported with the Welch correction

The results of the correlation analysis indicated that age and place of residence did not affect perceptions of individual aspects of HRQoL. Regarding education, only a weak correlation was found with the Eat, Weight, and Body domain (rho = 0.19; *p* = 0.006). On the other hand, the level of perceived pain showed significant correlations with all domains of HRQoL. Moderate correlations were noted in the Physical, Vitality & Emotion, Treatment Burden & Health Perceptions, and Respiratory domains. Conversely, weak correlations were found in the Eat, Weight, Body, and Digestion domains (Table 6). In each case, negative correlations were observed, indicating that as pain intensity increases, the HRQoL for patients with CF declines.

**Table 6:** Correlation analysis results for health-related quality of life domains and age, education level, place of residence, and perceived pain level

Domain	Age		Education level		Place of residence		Level of pain experienced	
	*r*	*p*	rho	*p*	rho	*p*	*r*	*p*
Physical	-0.11	0.109	-0.02	0.727	-0.11	0.090	-0.48	< 0.001
Vitality & Emotion	0.08	0.232	0.08	0.259	-0.06	0.350	-0.53	< 0.001
Treatment Burden & Health Perceptions	-0.10	0.153	0.01	0.875	0.02	0.716	-0.42	< 0.001
Respiratory	-0.10	0.155	-0.04	0.588	0.03	0.677	-0.41	< 0.001
Eat, Weight, Body	0.10	0.142	0.19	0.006	-0.02	0.802	-0.33	< 0.001
Digestion	0.03	0.607	0.08	0.232	-0.05	0.437	-0.28	< 0.001

We also analysed whether there were differences between the groups regarding their educational or professional situation over the past two weeks. Most patients (*n* = 126) attended school or work as usual. A significant number of patients were consistently inactive in terms of education or work (*n* = 55). The remaining patients missed school or work for health reasons (*n* = 25) or other reasons (*n* = 14). The comparison results indicated statistically significant effects on HRQoL in the domains of Physical, Treatment Burden & Health Perceptions, and Respiratory (strong effects), as well as in the domains of Vitality & Emotion and Eat, Weight, Body (moderate effects). No differences were found between the groups in the Digestion domain.

Post-hoc tests revealed that individuals who had attended school or work without any changes in the last two weeks reported a higher HRQoL than those who had missed school or work due to health reasons. A similar pattern was observed in comparison with individuals who were educationally or professionally inactive. Detailed results are presented in Table 7.

**Table 7:** Health-related quality of life domain results based on educational or professional situation over the past 2 weeks

Domain	Educational or professional situation over the last 2 weeks	Mean rank	Me	IQR	H(3)	*p*-value	η^2^
Physical	Attending school/work as usual	128.54^a^	34.00	9.00	30.81	< 0.001	0.14
	Absence from school/work for reasons other than health	127.79^ab^	34.00	9.25			
	Absence from school/work due to health reasons	76.52^b^	27.00	10.50			
	Educational/professional inactivity	80.22^b^	27.00	16.00			
Vitality & Emotion	Attending school/work as usual	123.13^a^	24.00	7.00	21.83	< 0.001	0.10
	Absence from school/work for reasons other than health	138.29^ac^	24.50	3.75			
	Absence from school/work due to health reasons	70.78^b^	19.00	6.00			
	Educational/professional inactivity	92.55^bc^	22.00	7.00			
Treatment Burden & Health Perceptions	Attending school/work as usual	131.45^a^	22.00	6.00	33.75	< 0.001	0.15
	Absence from school/work for reasons other than health	102.07^ab^	19.50	5.50			
	Absence from school/work due to health reasons	76.66^b^	18.00	4.00			
	Educational/professional inactivity	80.04^b^	18.00	6.00			
Respiratory	Attending school/work as usual	128.77^a^	21.00	5.00	32.67	< 0.001	0.15
	Absence from school/work for reasons other than health	129.93^ac^	22.00	4.00			
	Absence from school/work due to health reasons	73.06^b^	17.00	4.50			
	Educational/professional inactivity	80.71^bc^	18.00	8.00			
Eat, Weight, Body	Attending school/work as usual	123.92^a^	22.00	6.00	20.72	< 0.001	0.09
	Absence from school/work for reasons other than health	127.82^ac^	21.50	6.50			
	Absence from school/work due to health reasons	70.62^b^	17.00	6.50			
	Educational/professional inactivity	93.47^bc^	19.00	6.00			
Digestion	Attending school/work as usual	116.95	10.00	2.00	5.37	0.146	0.02
	Absence from school/work for reasons other than health	79.61	8.50	2.00			
	Absence from school/work due to health reasons	110.22	9.00	2.00			
	Educational/professional inactivity	103.72	9.00	3.00			

H(3) – Kruskal-Wallis test result; IQR – interquartile range; Me – median; η2 – eta squared

The mean ranks of groups labeled with different letters differ significantly (*p* < 0.05) according to the Dunn-Bonferroni post hoc test

## Discussion

In our study, we examined the HRQoL of Polish adult patients with CF and the various factors that affect it. As the availability of highly effective medications that significantly extend survival rates improves, it becomes increasingly important to analyse HRQoL in depth. The care provided to this patient group has undergone significant changes in recent years, with a growing focus on psychosocial and daily-life factors that influence their daily lives. In particular, growing attention has been paid to psychosocial aspects such as mental health and social support, which are increasingly recognized as important determinants of well-being in adults with CF [32, 33]. Despite recognition of this problem and the existence of recommendations for screening for generalized anxiety and depression, as well as psychological management, there remains a need to further expand and promote interventions that extend beyond routine medical care [34]. Additionally, broader life-course issues, including education and employment, have been shown to significantly influence HRQoL and treatment outcomes in this population [35, 36]. This makes our study particularly relevant, as it sheds light on factors that affect HRQoL and identifies areas for urgent improvement. Moreover, it’s worth emphasising that this study is the largest of its kind conducted among CF patients in Poland.

In a previous publication, we established the optimal factor structure of the tool for Polish adult patients, which we used to base our calculations [27]. Our findings reveal that participants obtained the lowest scores in two domains: Vitality & Emotion and Treatment Burden & Health Perceptions, with Vitality & Emotion being rated the lowest. It indicates that this patient group experiences limited vitality and that their emotional needs are not being fully addressed, which may also reflect the broader psychosocial challenges associated with living with a lifelong and relatively uncommon condition. Notably, the scores for emotion-related aspects in our study are considerably lower than those reported in other studies worldwide [37–40]. Interestingly, a relatively low score in this aspect was obtained in another Polish study conducted before and at the beginning of the COVID-19 pandemic [26, 41]. The markedly lower results in this domain are unexpected and warrant further analysis. Other studies worldwide consistently show that vitality is among the least positively rated areas, aligning with our results [42–44]. Various factors may contribute to the feelings of fatigue reported by this patient group. These factors include disease progression and exacerbations, limited capacity for an active lifestyle, and the presence of mood disorders [45–47]. The results of other studies also suggest that reduced energy levels in this patient population may be linked to systemic inflammation [48, 49]. Moreover, the burden of treatment and the demands of a therapeutic routine are likely contributing factors. This aligns with our findings, which identify Treatment Burden & Health Perceptions as the second-lowest-rated domain. The literature highlights treatment burden as a significant challenge within the CF patient community [50, 51]. This aspect deserves careful attention when devising treatment plans, emphasising the need for individualized interventions [52].

When examining the sociodemographic factors that influence HRQoL, it is worth highlighting disparities between women and men. In the domains of Physical, Vitality & Emotion, women reported a lower HRQoL than men. This difference may stem from a more severe progression of the disease among women [53–55]. Interestingly, men tended to give less favourable ratings within the Eat, Weight, Body domain. The present findings corroborate earlier research, which found that women frequently assess their experiences with weight and body image more positively [56–58]. Cultural influences may shape such assessments, as women might feel more satisfied with having a lower body weight and a slim figure. Conversely, men might express frustration regarding their thin physique and challenges in building muscle mass [59].

In our study, we noted only one statistically significant relationship between marital status and HRQoL. Patients in relationships scored higher on the Vitality & Emotion domain. This suggests that having support from a close person can significantly impact this aspect of HRQoL for adults with CF. The impact of social support is well established across research, with findings suggesting that its deficiency can result in poorer health outcomes and hinder daily functioning [60, 61].

Surprisingly, we did not find any statistically significant correlations between patients’ ages and their HRQoL. While other studies have shown that age can affect HRQoL, their findings have often been inconsistent and varied across different domains [62]. It appears that age is not a crucial factor in how Polish patients with CF perceive their HRQoL. The same applies to place of residence. This likely reflects the increasing urbanization of suburban areas and the resulting blurring of traditional distinctions, including sociodemographic aspects, between rural and urban populations, which may explain the lack of a clear relationship with patients’ HRQoL.

Interesting correlations were observed in patients’ educational and professional situations. Only a small percentage (25%) of patients were educationally or professionally inactive for an extended period, a figure significantly lower than those reported in studies worldwide [15]. This lack of educational and professional engagement may be linked to the patients’ health status and disease severity. Generally, individuals in better health and with less severe symptoms are more likely to pursue educational and professional opportunities than those who are less healthy [15]. In our study, individuals who were inactive in both education and professional life reported a lower HRQoL than their professionally active counterparts, a finding echoed by other studies [63, 64].

When delving into the health factors affecting the HRQoL for Polish adults living with CF, it’s essential to consider comorbidities. Many patients face additional health challenges, including diabetes, pancreatic insufficiency, chronic rhinosinusitis, depression, or generalised anxiety [65]. Our study revealed that patients dealing with additional conditions reported poorer outcomes in two key domains: Physical and Treatment Burden & Health Perceptions. Therefore, when evaluating a patient’s health, it is crucial to account not only for the primary disease but also for comorbidities, as these can significantly influence physical functioning and health perceptions. The impact of comorbidities on HRQoL should also be analysed further. Moreover, as the average age of patients has risen dramatically in recent years, this may lead to a greater prevalence of age-related diseases [66].

The use of CFTR modulators has positively influenced patients’ HRQoL. Those who utilised these treatments showed better outcomes in key areas, including Physical, Treatment Burden & Health Perceptions, Respiratory, and the Eat, Weight, Body domains. Other studies have also confirmed the significance of CFTR modulators for HRQoL. The most frequently analysed domain was Respiratory, which showed a significantly positive effect [67–69]. Some studies also examined effects across other domains and reported noteworthy improvements [70–73]. However, many did not observe improvements in the Digestion domain, a finding we also confirm in our study [71–73]. This highlights the need for special attention and targeted measures for those individuals who, for various reasons, are either not receiving or do not qualify for CFTR modulator treatments, as they require dedicated efforts to enhance their HRQoL.

Pain emerged as a key predictor of HRQoL in patients. Although the average pain levels reported were relatively low, they correlated significantly with all HRQoL domains. The existing literature highlights that pain can restrict physical activity and hinder the ability to perform daily tasks [74–76]. Additionally, it can adversely affect sleep, leading to interruptions that contribute to increased fatigue [77]. The chronic nature of pain can also pave the way for mental health issues, such as depression [78]. These factors cumulatively impact overall HRQoL, emphasising the need for clinicians to prioritise pain management by regularly assessing pain levels [16].

Our study has several limitations. Despite being the most extensive study conducted in Poland thus far and involving multiple centres, it cannot be deemed fully representative of the nation. This limitation arises from the way the sample was selected and the varying patient numbers across the facilities. Moreover, the cross-sectional design of the study prevented us from establishing causal relationships. The limitations of our study were also due to the method used to distribute questionnaires to patients. For this reason, we did not have access to medical records, so we could not collect reliable data on patients’ clinical status. Consequently, our analysis lacks crucial variables such as lung function and body mass index. Thus, we believe it is essential to pursue further research involving larger groups of Polish patients, including qualitative or mixed-methods research procedures, to gain deeper insights into the factors influencing HRQoL. Future studies should aim to include a thorough examination of health variables and their effects on HRQoL, and could be further expanded to incorporate psychosocial factors, such as coping strategies, illness perception, and social support.

## Conclusions

As adults with CF live longer, the importance of their overall HRQoL becomes increasingly significant. This study aimed to evaluate the HRQoL among adult CF patients in Poland and identify sociodemographic and health factors that influence it. The findings reveal that HRQoL in this population varies widely across assessed domains and various individual and clinical aspects.

Notably, CFTR modulators were found to have a positive impact on patients’ HRQoL. Therefore, it is crucial to involve as many patients as possible in these therapies. Special attention should be given to those who do not qualify for such treatments, and their HRQoL should be closely monitored. Additionally, participation in educational or professional activities was associated with improved HRQoL, underscoring the essential role of active social engagement. In contrast, the level of pain experienced by patients had a distinctly adverse effect on their HRQoL, emphasising the need for regular assessment and effective management of pain.

Other factors, such as gender, marital status, education, and comorbidities, influenced only certain domains of HRQoL. Interestingly, age and place of residence did not show any significant correlation with the CFQ-R results. This may suggest that, as treatment options evolve and patients’ overall health improves, traditional sociodemographic factors could be less pronounced, although certain associations may still be observed.

The results obtained have significant practical implications. They underscore the need for comprehensive, interdisciplinary care for adult patients with cystic fibrosis. This approach should integrate pharmacological treatment with psychological and social support to enhance their overall HRQoL.

## Supplementary Information

Below is the link to the electronic supplementary material.

Supplementary Material 1

## Data Availability

The datasets generated during and/or analyzed during the current study are available from the corresponding author on reasonable request.

## Declarations

### Declaration of AI use

During the preparation of this manuscript, the authors used ChatGPT-5 to improve language clarity and grammar. All content was critically reviewed and verified by the authors to ensure accuracy and integrity. The authors reviewed and edited the output and take full responsibility for the content of this publication.
