# Supplementary material for: Determinants of health-related quality of life in adults with cystic fibrosis: the role of selected sociodemographic and treatment-related factors
Source: Orphanet J Rare Dis. 2026 Apr 24;21:220. doi: 10.1186/s13023-026-04362-x (PMC13277195; doi:10.1186/s13023-026-04362-x)
Supplement: Supplementary file 1 — Supplementary Material 1 [file 13023_2026_4362_MOESM1_ESM.docx]

**Table S1. Descriptive statistics along with the result of the Kolmogorov–Smirnov test for the domains of the original CFQ-R 14+**

| Domain | M | Me | SD | Sk | Kurt | Min | Max | D | p |
| --- | --- | --- | --- | --- | --- | --- | --- | --- | --- |
| **Raw scores** |  |  |  |  |  |  |  |  |  |
| Physical | 24,78 | 26,00 | 6,19 | -0,88 | 0,07 | 8,00 | 32,00 | 0,14 | **<0,001** |
| Vitality | 10,44 | 11,00 | 2,67 | -0,09 | -0,38 | 4,00 | 16,00 | 0,10 | **<0,001** |
| Emotion | 14,09 | 14,00 | 3,63 | -0,36 | -0,58 | 5,00 | 20,00 | 0,10 | **<0,001** |
| Eat | 10,54 | 12,00 | 1,96 | -1,31 | 1,02 | 3,00 | 12,00 | 0,28 | **<0,001** |
| Treatment Burden | 9,05 | 9,00 | 1,99 | -0,48 | -0,01 | 3,00 | 12,00 | 0,13 | **<0,001** |
| Health Perceptions | 8,25 | 8,00 | 2,23 | -0,16 | -0,40 | 3,00 | 12,00 | 0,09 | **<0,001** |
| Social | 17,70 | 18,00 | 3,68 | -0,37 | -0,30 | 7,00 | 24,00 | 0,09 | **<0,001** |
| Body | 8,71 | 9,00 | 2,41 | -0,32 | -0,72 | 3,00 | 12,00 | 0,12 | **<0,001** |
| Role | 12,25 | 12,00 | 2,36 | -0,45 | -0,09 | 5,00 | 16,00 | 0,11 | **<0,001** |
| Weight | 3,15 | 4,00 | 1,06 | -0,88 | -0,61 | 1,00 | 4,00 | 0,32 | **<0,001** |
| Respiratory | 19,18 | 20,00 | 4,05 | -0,70 | -0,47 | 8,00 | 24,00 | 0,15 | **<0,001** |
| Digestion | 9,60 | 10,00 | 1,90 | -0,80 | 0,55 | 3,00 | 12,00 | 0,14 | **<0,001** |
| **Transformed scores** |  |  |  |  |  |  |  |  |  |
| Physical | 69,91 | 75,00 | 25,81 | -0,88 | 0,07 | 0,00 | 100,00 | 0,14 | **<0,001** |
| Vitality | 53,64 | 58,33 | 22,27 | -0,09 | -0,38 | 0,00 | 100,00 | 0,10 | **<0,001** |
| Emotion | 60,58 | 60,00 | 24,18 | -0,36 | -0,58 | 0,00 | 100,00 | 0,10 | **<0,001** |
| Eat | 83,79 | 100,00 | 21,82 | -1,31 | 1,02 | 0,00 | 100,00 | 0,28 | **<0,001** |
| Treatment Burden | 67,27 | 66,67 | 22,14 | -0,48 | -0,01 | 0,00 | 100,00 | 0,13 | **<0,001** |
| Health Perceptions | 58,33 | 55,56 | 24,83 | -0,16 | -0,40 | 0,00 | 100,00 | 0,09 | **<0,001** |
| Social | 65,03 | 66,67 | 20,42 | -0,37 | -0,30 | 5,56 | 100,00 | 0,09 | **<0,001** |
| Body | 63,43 | 66,67 | 26,73 | -0,32 | -0,72 | 0,00 | 100,00 | 0,12 | **<0,001** |
| Role | 68,75 | 66,67 | 19,65 | -0,45 | -0,09 | 8,33 | 100,00 | 0,11 | **<0,001** |
| Weight | 71,67 | 100,00 | 35,33 | -0,88 | -0,61 | 0,00 | 100,00 | 0,32 | **<0,001** |
| Respiratory | 73,23 | 77,78 | 22,50 | -0,70 | -0,47 | 11,11 | 100,00 | 0,15 | **<0,001** |
| Digestion | 73,28 | 77,78 | 21,09 | -0,80 | 0,55 | 0,00 | 100,00 | 0,14 | **<0,001** |

D – Kolmogorov-Smirnov test result; Kurt – kurtosis; M – mean; Max – maximum value; Me – median; Min – minimum value; SD – standard deviation; Sk – skewness.
